# Research Progress of the Functional Role of ACK1 in Breast Cancer

**DOI:** 10.1155/2019/1018034

**Published:** 2019-10-20

**Authors:** Xia Liu, Xuan Wang, Lifang Li, Baolin Han

**Affiliations:** ^1^Department of Radiotherapy, Tianjin Baodi Hospital, Baodi Clinical College of Tianjin Medical University, Tianjin 301800, China; ^2^Department of Critical Care Medicine, Tianjin Baodi Hospital, Baodi Clinical College of Tianjin Medical University, Tianjin 301800, China

## Abstract

ACK1 is a nonreceptor tyrosine kinase with a unique structure, which is tightly related to the biological behavior of tumors. Previous studies have demonstrated that ACK1 was involved with multiple signaling pathways of tumor progression. Its crucial role in tumor cell proliferation, apoptosis, invasion, and metastasis was tightly related to the prognosis and clinicopathology of cancer. ACK1 has a unique way of regulating cellular pathways, different from other nonreceptor tyrosine kinases. As an oncogenic kinase, recent studies have shown that ACK1 plays a critical regulatory role in the initiation and progression of tumors. In this review, we will be summarizing the structural characteristics, activation, and regulation of ACK1 in breast cancer, aiming to deeply understand the functional and mechanistic role of ACK1 and provide novel therapeutic strategies for breast cancer treatment.

## 1. Introduction

Breast cancer is the most common type of cancer and the leading cause of cancer-related death among women worldwide [1]. Although modern medicine for comprehensive treatment of breast cancer has improved a lot with a reduced mortality rate, however, the prevention and treatment of breast cancer remain problematic. Triple-negative breast cancer still lacks effective drug treatment today [2]. Breast cancer research found that many ACK1 tyrosine kinase signaling proteins in many tumor cells are activated repeatedly [3–6]. ACK1 expression is positively correlated with the severity of the disease progression and negatively correlated with the survival rate in breast cancer patients [7, 8].

As a nonreceptor tyrosine kinase (or cytoplasmic tyrosine kinase), ACK1 does not receive signals directly from outside the cell but is activated quickly. Its activation is tightly regulated by receptor tyrosine kinases' activation [9–11]. The process is tightly and dynamically controlled by a series of the single signal paths or multiple phosphorylation cascades and forms tyrosine kinase connection [3, 12]. These signaling processes are dysfunctional during accelerated growth and differentiation of cells. It has been found that the overexpression of ACK1 is related to various tumors, including lung, prostate, stomach, pancreatic, breast, and ovarian cancers [8, 12–16]. Therefore, ACK1 plays a significant role in tumors, but the mechanism of activation and regulation in these tumors is not the same. This review summarizes the function and mechanism of ACK1 in breast cancer, aiming to deeply understand the relationship between ACK1 and breast cancer and providing a basis for personalized treatment of breast cancer.

## 2. Structure and Function of ACK1

Human ACK1 is a 120 kDa protein that contains 1038 amino acid residues [3, 17]. Its coding gene TNK2 is located in the region of chromosome 3q29 [6]. ACK1 plays a role based on its unique structural characteristics, and it contains many essential domains related to its functions. The biological functions of some domains have been reported. For instance, the SAM domain was involved with the membrane localization, dimerization, and activation of ACK1 [18, 19]. The CRIB domain mediates the interaction between ACK1 and Cdc42 [3, 20], and the PPXY motif mediates the interaction between ACK1 and WW domain [21]. The MHR domain mediates the interaction between ACK1 and receptor tyrosine kinase [22]. The UBA domains are involved with the regulation of ACK1 binding to ubiquitin and its polyubiquitination and degradation [23].

## 3. Activation and Degradation of ACK1 in Breast Cancer Cells

ACK1 high expression is closely related to the progress of breast cancer. ACK1 kinase domain interaction with the downstream SH3, CRIB, proline-rich sequence, and MHR domain to affect its kinase activity. A pathological condition characterized by the activation of ACK1, or excessive expression, mainly with three ways of ACK1 activation [24]: (1) Just like a variety of other receptor tyrosine kinases, ACK1 goes through protein interaction and then activates itself. Cells treated with growth factors showed not only rapid activation of their respective RTK, but also activation of ACK1 through tyrosine phosphorylation [7, 25, 26]. This phenomenon suggested that multiple RTKs may potentially interact with ACK1 to cause its activation. (2) The upregulation of the ACK1 gene results in increased mRNA and protein level, which further promotes its dimerization and activation. This process serves as another activation mechanism independent of the RTK-regulated activation in many cancer types. The upregulation of ACK1 has been previously observed in various cancer types such as cervical, ovarian, lung, head and neck squamous cell, breast, prostate, and stomach cancers [6, 7, 15, 27–30]. (3) The mutation results in abnormal activation of ACK1, which can be activated by disinhibition. Among them, four missense mutations, R34L, R99Q, E346K, and M409I, were evidently reported to be located in different regions of ACK1 [5, 7]. The ACK1-E346K mutation was the first to be identified in ovarian cancer with a significant increase in ACK1 self-activation [7, 31, 32].

However, in breast cancer, ACK1 gene upregulation (3.4%) and somatic automatic activation mutation (0.1%) are relatively rare, and their activation is mainly by the interaction between RTK and ACK1 [33]. Recent studies have shown that several ubiquitination enzymes, including NEDD4-1 [21], NEDD4-2, SIAH1, and SIAH2 [34], can ubiquitinate ACK1 and induce ACK1 degradation [23, 34]. SIAH2 may be a target gene of E2/ER (estrogen receptor) and regulate the ubiquitylation and degradation of ACK1. In ER-positive breast cancer cells, the cells can activate the ER and estrogen and then ubiquitin ACK1 and reduce the ACK1 level. The lack of ER may increase the stability of ACK1, and in the absence of estrogen, breast cancer cells continually express ACK1. Therefore, it is very important to promote the survival and metastasis of breast cancer.

## 4. Biological Behavior of ACK1 in Breast Cancer Cells

The biological behavior of ACK1 in breast cancer cells is mainly manifested as promoting the growth and proliferation of tumor cells and promoting the metastasis and invasion of tumor cells. ACK1 is of great significance for the survival of cancer cells. ACK1 promotes cell survival by actively regulating survival pathways to prevent cell death [6]. Previous studies have found that the phosphorylation of ACK1 may be related to the progression of breast cancer [3, 7]. However, the functional signals expressed by ACK1 and their role in the biological behavior of breast cancer have not been well elucidated.

## 5. ACK1 Promotes the Proliferation of Breast Cancer Cells

Most mammalian cells experience cell cycle arrest, followed by apoptosis, a process that is out of control and leads to tumor development [35, 36]. Yorkie, a transcription coactivator, promotes the transcription of proliferation and antiapoptotic genes, with which ACK1 can interact to promote tissue overgrowth [37]. Besides, ACK1 S985N mutation promotes cell proliferation, migration, and epithelial-mesenchymal transition [6]. Protein kinase AKT plays a central role in the cell growth, proliferation, and cell survival; AKT activation occurs in the ligand combined with RTK and promotes AKT transport to the plasma membrane [38]. Past researches mainly focus on RTK-mediated PI3K/AKT activation, and recent studies have found that RTK/ACK1/AKT signaling pathway is independent of the path of PI3K in regulating AKT activation. Around a third of the breast cancer showed AKT abnormal activation, and ACK1 promotes cell growth and proliferation by interacting with AKT [4, 6, 39].

## 6. ACK1 Is Involved in the Metastasis and Invasion of Breast Cancer Cells

ACK1 can enhance the migration and invasion ability of breast cancer cells by strengthening the EGFR signaling pathway [40]. ACK1 enhances cancer-causing epidermal growth factor (EGFR) signaling and has been shown to increase proliferation and invasiveness of breast cancer cells [41, 42]. Clinically, more than 20% of breast cancer patients are diagnosed with positive human epidermal growth factor receptor (EGFR), which is associated with a reduced survival rate of breast cancer [43, 44]. The effect of ACK1 on the EGFR signaling pathway has been demonstrated to promote cell migration by activating CDC42. Jillian Howlin et al. observed that the significant decrease of EGFR on the cell surface knocked down by ACK1 was caused by the parallel reduction in the migration ability of breast cancer cells [42]. The primary role of EGFR activation in breast cancer cells is to stimulate exercise, and it has been demonstrated for the first time that maintaining the ability of EGFR cell surface expression through ACK1 can enhance the invasion ability of breast cancer cells. Meanwhile, ACK1 inhibits the invasion of breast cancer cells by regulating BCAR1, but the mechanism remains unclear [42].

## 7. ACK1 Serves as a Marker for Diagnosis and Prediction of Breast Cancer

ACK1 is phosphorylated with tyrosine and interacts with many protein substrates to regulate critical cellular processes [3]. Previous studies have found that phosphorylation of ACK1 may be associated with breast cancer progression; ACK1 has specificity in phosphorylation that affects signal transmission at different sites [3, 7]. Most of its phosphorylation sites are unique, and this property is caused by the unusual substrate binding ability of ACK1 [45]. Detection of its tyrosine phosphorylation will help in the diagnosis, treatment, and prognosis of breast cancer.

The phosphorylation levels of ACK1's Tyr284-phosphorylation and AKT's Tyr176 phosphorylation are positively correlated with the severity of disease progression and negatively correlated with the survival rate of breast cancer patients. A significant increase in phosphorylation of ACK1-Tyr284 is a marker of ACK1 activation [7, 12, 26, 46], and this increased ACK1 activation is associated with poor tumor prognosis. In addition, the detection of p-Tyr176-AKT level in tumor biopsy can be used as an auxiliary diagnostic tool for personalized treatment with ACK1 inhibitors. ACK1 inhibitor combinations have been shown to benefit pancreatic, lung, breast, and prostate cancers that exhibit robust AKT Tyr176 phosphorylation [6, 12]. AKT phosphorylation at Ser473 phosphorylation (or thr308 phosphorylation) evaluates AKT activation and is generally evaluated as a positive outcome that can be treated with an inhibitor. PY518 phosphorylation is increased in triple-negative breast cancer cells [47]. Current studies have shown that ACK1 is not only hyperphosphorylated but also overexpressed in many highly aggressive triple-negative and triple-negative breast cancer cell lines and that ACK1 expression is associated with aggressive phenotypes in triple-negative breast cancer cell lines. AKT Tyr176 phosphorylation is abnormal in triple-negative breast cancer cell lines, which is sensitive to the ACK1 inhibitor (R-9BMS). Treating with ACK1 inhibitor (R-9BMS) can affect the proliferation of TNBCs, and through the detection of tyrosine phosphorylation will provide help for the diagnosis, treatment, and prognosis of breast cancer.

## 8. ACK1 and Endocrine Therapy for Breast Cancer

Breast cancer is a kind of heterogeneous disease. Endocrine therapy is an important means of comprehensive treatment of breast cancer in addition to surgery. The advantage of ER expression in breast cancer cells and the cell dependence on estrogen make tamoxifen successful in the treatment of ER-positive breast cancer, which can reduce the recurrence of breast cancer by nearly 50% [48]. Although most breast tumors initially respond well to tamoxifen therapy, most women develop tamoxifen resistance at approximately 15 months to 5 years [49]. Despite intensive research, the molecular mechanisms of tamoxifen resistance remain unclear. Drug resistance is a major clinical problem in breast cancer patients, and an in-depth understanding of this phenomenon will significantly help HER2-positive patients. Mahajan et al. demonstrated that ACK1 regulated HOXA1 expression and gave tamoxifen resistance by regulating the epigenetic activity of ER coactivator KDM3A without E2 [33]. Homeobox A1 (HOXA1) gene is a potent oncogene, and the active expression of HOXA1 is enough to cause the oncogenic transformation of human breast epithelial cells and has the ability of invasive tumor [50]. The expression of HOXA1 was significantly increased in regulatory therapy but was significantly downregulated in MCF-7 breast cancer cells treated with ACK1 inhibitor AIM-100 and dasatinib. The combined regulatory activity of ER mediated by ACK1 is crucial for promoting the transcription of HOXA1 gene. In the absence of estrogen, the regulatory protein ACK1 activates the Tyr-1114 phosphorylation site in the ER coactivator KDM3A. ER target gene transcription is promoted in estrogen-deficient environments, such as HOXA1. This may be a novel molecular mechanism for the acquisition of tamoxifen resistance in breast tumors with overexpression of HER2. This provides a new method for endocrine therapy of breast cancer patients; that is, ACK1 inhibitor AIM-100 or dasatinib inhibits ACK1 signal to alleviate the upregulation of drug resistance by HOXA1 in breast cancer patients [5]. ACK1 inhibitors have become a potential treatment for antihormone therapy of tumors in a variety of mechanisms that promote ACK1 activation in breast cancer [6]. This approach to personalized drug therapy may be beneficial for patients with tamoxifen-resistant breast cancer, revealing ACK1 inhibitor treatments such as dasatinib therapy, which is already an FDA-approved drug as an adjuvant treatment regimen.

A recent study found ACK1 regulation by AR signal is the critical mechanism; the ACK1 expression increased in quite several prostate cancer samples and ACK1 tyrosin284 phosphorylation also significantly increased, and the ACK1 activation was associated with poor prognosis of tumors. ACK1 Tyr284 phosphorylation and AR Tyr267 phosphorylation were positively correlated with the severity of the disease progression [7, 26, 52]. ACK1 phosphorylates AR and then promotes transcriptional activation at the target promoter. Activated ACK1/pTyr267-AR complex was recruited into ATM (ataxia telangiectasia mutant kinase) [46]. ATM is a regulator of DNA damage and cell cycle checkpoints signal pathway, ensuring the integrity of genes in cells to respond to DNA double-strand breaks [53]. In the absence of androgen, the Tyr267 phosphorylation of AR can promote ATM transcription, and studies have shown that increased expression of ATM protein and upregulation of genes related to maintenance of gene integrity may prevent the death of CPRC tumor cells [46]. Therefore, inhibition of ACK1-AR signaling, thereby inhibiting ACK1-mediated ATM levels, maybe a new therapeutic strategy for CRPC tumors, which often exhibits radiation resistance. The main downstream effector of ACK1 is AR, and both breast and prostate cancers are hormone-regulated cancers, indicating the potential of ER as another hormone receptor that interacts with ACK1, possibly making breast cancer cells more sensitive to radiotherapy by inhibiting ACK1-ER signaling.

Approximately 15–20% of breast cancers do not express the estrogen receptor, progesterone receptor, or HER2 receptor, collectively known as triple-negative breast cancer (TNBC) [54]. ER-positive breast cancer or HER2 cationic breast cancer can be treated with endocrine therapy or HER2-targeted therapy [55, 56]. Compared with other types of breast cancer, these tumors are usually aggressive and lack effective targeted treatment. There is currently no targeted therapy for TNBCs patients [57, 58]. But the nonreceptor tyrosine kinase ACK1 is activated in most aggressive TNBC cell lines. Wu et al. found that inhibiting the ACK1 signal not only reduced the proliferation of TNBC cells but also promoted the invasiveness of tumor formation in xenograft mice [8]. This phenomenon indicates the dependence of TNBCs on ACK1 signal in proliferation and invasion ability. In high-level basal-like breast cancer, the high level of ACK1 expression is closely related to poor prognosis of patients [59]. It is suggested that ACK1 is a new potential therapeutic target for TNBC. The loss of ACK1 causes the death of resistant cells to the EGFR inhibitor gefitinib [33]. Therefore, combining the inhibition of EGFR and ACK1 may be a new chemotherapy strategy to overcome resistance to gefitinib [14, 60]. Combining anti-ACK1 therapy with doxorubicin therapy in the treatment of invasive TNBCs provides a pathway for future targeted therapies based on breast cancer.

## 9. Conclusion

To sum up, ACK1 is activated in a variety of tumors by tyrosine phosphorylation of a range of proteins, particularly those essential for cell survival, growth, and proliferation and to regulate the activity [5, 27]. To date, it has been found that ACK1 interacts with a variety of receptor tyrosine kinases (EGFR), oncoproteins (AKT), tumor suppressor proteins (Wwox), and epigenetic modification regulatory proteins (KDM3A) in breast cancer [5, 6, 11, 33, 40]. Its overactivation mainly plays a vital role in the occurrence and development of breast cancer through downstream substrates. A better understanding of ACK1 signal pathway will reveal its participation in specific cell-signaling pathways for promoting growth and inhibiting apoptosis. ACK1 inhibitor drugs will have a broad prospect of clinical application, and at the same time, ACK1 Y284 phosphorylation as a marker in some breast cancer and pTyr-1114 KDM3A antibodies also has a significant clinical diagnostic value which can be used in patients for ACK1-positive breast cancer screening. However, it is not clear whether there are other mechanisms in ACK1-related tumors to promote the growth, proliferation, migration, and invasion of cancer cells through ACK1. Moreover, more ACK1 interaction proteins or substrates need to be further identified to better utilize them for personalized diagnosis and treatment of breast cancer.
